# Supplementary material for: Fungal Contamination of Building Materials and the Aerosolization of Particles and Toxins in Indoor Air and Their Associated Risks to Health: A Review
Source: Toxins (Basel). 2023 Feb 25;15(3):175. doi: 10.3390/toxins15030175 (PMC10054896; doi:10.3390/toxins15030175)
Supplement: Supplementary file 1 [file toxins-15-00175-s001.zip › toxins-2218741-supplementary.pdf]

Review

Fungal Contamination of Building Materials and the Aerosolization of Particles and Toxins in Indoor Air and their Associated Risks to Health: A Review

Mohamad Al Hallak, Thomas Verdier, Alexandra Bertron, Christine Roques and Jean-Denis Bailly

Table S1. Fungal species detected on surfaces of materials in different types of buildings according to sampling method, material type, method used for analysis and location of the study.

Type of building	Sampling method	Surface type	Analysis method	Species detected	Location [Ref]
**Damp and/or mouldy dwellings (no respiratory problems for occupants)**					
30 mouldy homes *	Swab on a 25 cm² area	Gypsum, wallpaper	DNA sequencing (ITS)	*Alternaria alternata*	France [75]
				Aspergillus versicolor	
				Cladosporium sphaerospermum	
				Penicillium chrysogenum	
				Stachybotrys chartarum	
32 mouldy dwellings *	Swab on a 25 cm² area	Wallpaper	Morphological identification after culturing	*Aspergillus versicolor*	France [76]
				Aspergillus niger	
				Aspergillus fumigatus	
				Cladosporium cladosporioides	
				Cladosporium sphaerospermum	
				Penicillium chrysogenum	
				Ulocladium chartarum	
34 mouldy dwellings	Swab on a 25 cm² area	Wallpaper	Morphological identification after culturing	*Aspergillus flavus*	Poland [77]
				Aspergillus niger	
				Aspergillus versicolor	
				Penicillium chrysogenum	
				Stachybotrys Chartarum	
Mouldy buildings	Contact plates	Plaster, concrete, wallpaper, gypsum		*Alternaria tenuissima*	Denmark [78]
				Aspergillus fumigatus	

		& wood	Morphological identification after culturing	*Aspergillus niger*<br>*Aspergillus versicolor*<br>*Aspergillus wentii*<br>*Cladosporium sphaerospermum*	
22 mouldy dwellings	Scraping:<br>1.5 grams/ sample	Wallpaper	Morphological identification after culturing	*Acremonium charticola*<br>*Aspergillus versicolor*<br>*Cladosporium cladosporioides*<br>*Penicillium chrysogenum*	Poland [79]
Mouldy storage room in a family house *	Swab on a 25 cm^2 area	Wood	DNA sequencing (ITS)	*Aspergillus penicillioides*<br>*Aspergillus subversicolor*<br>*Stachybotrys echinata*<br>*Sterigmatomyces halophilus*	USA [80]
60 mouldy dwellings	Swab on a 25 cm^2 area	Wallpapers, Wood	DNA sequencing (ITS)	*Aspergillus unguis*<br>*Cladosporium halotolerans*<br>*Cyberlindnera jadinii*	USA [67]
32 mouldy homes *	Swab on a 25 cm^2 area	Wallpaper, ceramic and wood	DNA sequencing (ITS)	*Alternaria soliaridae*<br>*Aspergillus niger*<br>*Aspergillus piperis*<br>*Aspergillus versicolor*<br>*Cladosporium halotolerans*<br>*Cladosporium sphaerospermum*<br>*Penicillium aurantiogriseum*<br>*Penicillium oxalicum*<br>*Stachybotrys echinata*	USA [81]
3 Mouldy homes	Swab on a 25 cm^2 area	Brick walls	DNA sequencing (ITS) & morphological identification after culturing	*Acremonium charticola*<br>*Aspergillus canadensis*<br>*Aspergillus versicolor*<br>*Cladosporium sphaerospermum*<br>*Debaryomyces hansenii*<br>*Monocillium tenue*	Denmark [82]

				Penicillium roseopurpureum	
				Verrucocladosporium dirinae	
24 mouldy houses	Not mentioned	Wallpapers, plasters, wood	Morphological identification after culturing	*Acremonium* spp	Germany [83]
				Aspergillus calidoustus	
				Aspergillus fumigatus	
				Aspergillus niger	
				Aspergillus versicolor	
				Chaetomium spp	
				Cladosporium spp	
				Fusarium spp	
				Penicillium spp	
				Stachybotrys spp	

Type of building	Sampling method	Surface type	Analysis method	Species detected	Location [Ref]
**Dwellings with respiratory problems for occupants (no visible mould development)**					
100 dwellings with occupants with respiratory diseases	Contact plates	Wallpaper	Morphological identification after culturing	*Aspergillus glaucus*	France [73]
				Aspergillus niger	
				Aspergillus versicolor	
				Cladosporium sphaerospermum	
				Stachybotrys chartarum	
27 dwellings with occupants with respiratory diseases *	Swab on a 25 cm^2 area	Wallpaper	Morphological identification after culturing	*Aspergillus fumigatus*	France [76]
				Aspergillus niger	
				Aspergillus versicolor	
				Cladosporium cladosporioides	
				Cladosporium sphaerospermum	
				Penicillium chrysogenum	
				Ulocladium chartarum	
25 homes with occupants with respiratory diseases *	Swab on a 25 cm^2 area	Gypsum, wallpaper	DNA sequencing (ITS)	*Alternaria alternata*	France [75]
				Aspergillus versicolor	
				Cladosporium sphaerospermum	
				Penicillium chrysogenum	
				Stachybotrys chartarum	
	Contact plate	Wallpaper		*Aspergillus flavus*	France [84]

90 dwellings with occupants with respiratory diseases *			Morphological identification after culturing	*Aspergillus fumigatus*<br>*Aspergillus niger*<br>*Aspergillus versicolor*	

Type of building	Sampling method	Surface type	Analysis method	Species detected	Location [Ref]
**Mouldy or damp dwellings where occupants suffer from respiratory problems**					
One mouldy building with respiratory health problems for occupants *	Scraping (Stripping wallpaper)	Wallpaper	Morphological identification after culturing	*Aspergillus niger*<br>*Aspergillus sydowii*<br>*Stachybotrys chartarum*<br>*Ulocladium chartarum*	USA [85]
5 mouldy buildings with respiratory health problems for occupants	Swab on a 25 cm^2 area	Plaster, Wallpaper, wood.	Morphological identification after culturing	*Aspergillus niger*<br>*Aspergillus ustus*<br>*Penicillium aurantiogriseum*<br>*Stachybotrys chartarum*	USA [9]
12 mouldy dwellings with respiratory health problems for occupants	Swab on a 25 cm^2 area	Wallpaper, wood.	DNA sequencing (β-tubulin)	*Acremonium strictum*<br>*Aspergillus flavus*<br>*Aspergillus fumigatus*<br>*Aspergillus sydowii*<br>*Aspergillus versicolor*<br>*Chaetomium murarum*<br>*Cladosporium sphaerospermum*	Belgium [86]

				Ulocladium botrytis	
1012 mouldy dwellings with respiratory health problems for occupants *	Swab on a 25 cm^2 area	Wallpaper	Morphological identification after culturing	*Acremonium* spp	France [72]
				Alternaria alternata	
				Aspergillus flavus	
				Aspergillus fumigatus	
				Aspergillus niger	
				Aspergillus ochraceus	
				Aspergillus versicolor	
				Cladosporium spp	
				Penicillium spp	
				Stachybotrys spp	

Type of building	Sampling method	Surface type	Analysis method	Species detected	Location [Ref]
**Normal buildings (no dampness, no visible mould and no respiratory problems for occupants)**					
31 dwellings	Swab on a 25 cm^2 area	Walls and ceilings	Morphological identification after culturing	*Aspergillus niger*	USA [87]
				Aspergillus versicolor	
				Stachybotrys chartarum	
59 dwellings *	Swab on a 25 cm^2 area	Wallpaper	Morphological identification after culturing	*Aspergillus fumigatus*	France [76]
				Aspergillus niger	
				Aspergillus versicolor	
				Cladosporium cladosporioides	
				Cladosporium sphaerospermum	
				Penicillium chrysogenum	
				Ulocladium chartarum	
55 control homes *	Swab on a 25 cm^2 area	Gypsum, wallpaper	DNA sequencing (ITS)	*Alternaria alternata*	France [75]
				Aspergillus versicolor	
				Cladosporium sphaerospermum	
				Penicillium chrysogenum	
				Stachybotrys chartarum	
10 buildings *	Scraping	Plasters, ceilings	Morphological identification after culturing	*Alternaria alternata*	Slovakia [66]
				Aspergillus flavus	
				Aspergillus parasiticus	
				Cladosporium herbarum	
				Cladosporium sphaerospermum	
				Fusarium verticillioides	
				Penicillium verrucous	

51 rooms in university	Passive petri plate gravitational dust settling method	Gypsum boards	DNA sequencing (ITS) & morphological identification after sampling	*Alternaria alternata*	Canada [88]
				Aspergillus fumigatus	
				Aspergillus niger	
				Aspergillus versicolor	
				Cladosporium spp	
				Penicillium spp	
				Stachybotrys chartarum	
35 homes *	Swab on a 25 cm² area	Wallpaper, ceramic and wood	DNA sequencing (ITS)	*Alternaria soliaridae*	USA [81]
				Aspergillus niger	
				Aspergillus piperis	
				Aspergillus versicolor	
				Cladosporium halotolerans	
				Cladosporium sphaerospermum	
				Penicillium aurantiogriseum	
				Penicillium oxalicum	
				Stachybotrys echinata	
9 homes	Swab on a 25 cm² area	Wood	DNA sequencing (ITS) & Morphological identification after culturing	*Acremonium charticola*	Denmark [82]
				Aspergillus domesticus	
				Aspergillus glaucus	
				Aspergillus versicolor	
				Aspergillus vitricola	
				Cladosporium allicinum	
10 higher-education institutes (HEI)	Swab on a 25 cm² area	Wallpapers, wood	Morphological identification after culturing	*Cladosporium cladosporioides*	Portugal [63]
				Penicillium brevicompactum	
				Penicillium chrysogenum	
				Saccharomyces cerevisiae	
				Stachybotrys chartarum	
				Aspergillus fumigatus	
				Aspergillus niger	
				Chrysonilia sitophila	
				Cladosporium spp	
				Penicillium spp	

Type of building	Sampling method	Surface type	Analysis method	Species detected	Location [Ref]
**Hospitals**					
19 patient rooms in a hospital *	Swab on a 25 cm² area	Wallpapers, bed-plastic,	Morphological identification after culturing	*Aspergillus flavus*	France [89]
				Aspergillus fumigatus	
				Aspergillus niger	
2420 patients' rooms in 3 hospitals *	Swab on a 25 cm² area	Sinks, tables, wallpapers	Morphological identification after culturing	*Aspergillus flavus*	Greece [90]
				Aspergillus fumigatus	
				Aspergillus niger	
				Candida albicans	
15 patient rooms in a hospital *	Contact plates (Bio contact applicator)	Wallpapers	Morphological identification after culturing	*Aspergillus flavus*	France [91]
				Aspergillus fumigatus	
				Aspergillus glaucus	
				Aspergillus hiratsukae	
				Aspergillus niger	
				Aspergillus terreus	
				Aspergillus versicolor	
4 elderly care centers *	Swab on a 25 cm² area	Wallpaper	Morphological identification after culturing	*Aspergillus candidus*	Portugal [64]
				Aspergillus fumigatus	
				Aspergillus niger	

Type of building	Sampling method	Surface type	Analysis method	Species detected	Location [Ref]
**Others**					
36 wine cellars *	Swab on a 25 cm^2 area	Walls (brick or concrete) and wine barrels	Morphological identification after culturing	*Absidia macrospora*	Austria [92]
				Alternaria alternata	
				Aspergillus fumigatus	
				Aspergillus niger	
				Aspergillus versicolor	
				Cladosporium cladosporioides	
				Cladosporium sphaerospermum	
				Fusarium solani	
				Penicillium chrysogenum	
				Penicillium olsonii	
				Wallemia sebi	
				Zasmidium cellare	
3 archives buildings *	Swab on a 25 cm^2 area	Wallpaper, wood	Morphological identification after culturing	*Aspergillus fumigatus*	Portugal [93]
				Aspergillus niger	
				Aspergillus versicolor	
				Stachybotrys chartarum	
6 museums *	Swabbing on a 25 cm^2 area and by using contact plates samples	Shelves, walls and historical objects	Morphological identification after culturing	*Aspergillus flavus*	Poland [94]
				Aspergillus fumigatus	
				Aspergillus niger	
				Aspergillus ochraceus	
				Aspergillus parasiticus	
				Aspergillus versicolor	

				Cladosporium herbarum	
				Penicillium carheum	
Museum *	Swab on a 25 cm^2 area	Wood	Morphological identification after culturing	*Aspergillus ustus*	Slovakia [95]
				Aspergillus versicolor	
				Chaetomium globosum	
				Penicillium citrinum	
				Penicillium copticola	
				Penicillium sclerutiorum	
				Penicillium spinulosum	

*: Studies in which both air sampling and surface sampling were carried out
